# Comparing lactic acid bacteria biodiversity in irritable bowel syndrome and healthy gut microbiota

**DOI:** 10.1002/fsn3.4477

**Published:** 2024-09-30

**Authors:** Aysooda Azimi, Masoud Yavarmanesh, Mehran Gholamin

**Affiliations:** ^1^ Department of Food Science and Technology, Faculty of Agriculture Ferdowsi University of Mashhad Mashhad Iran; ^2^ Department of Laboratory Sciences, School of Paramedical Sciences Mashhad University of Medical Sciences Mashhad Iran

**Keywords:** 16S rRNA, gut microbiota, irritable bowel syndrome, lactic acid bacteria, phylogenetic tree

## Abstract

Irritable bowel syndrome (IBS) is a prevalent gut disorder linked to changes in the gut microbiota, including lactic acid bacteria (LAB). However, research on LAB biodiversity in IBS patients is limited. This study aimed to compare LAB microbiota in healthy individuals and those with IBS through biochemical and molecular techniques. Fecal samples from 15 IBS patients and 13 healthy individuals were collected, and LAB were isolated using biochemical methods. Fifty isolates were chosen based on Gram staining and catalase tests and identified through 16S rRNA gene sequencing. A phylogenetic tree was used to analyze strain diversity, and correlation diagrams and swarm plots were employed to explore variable relationships. The study revealed a significant difference in LAB numbers between IBS and healthy subjects, with average of 5.91 and 6.63, respectively. Most bacteria were Gram‐positive cocci or bacilli, with homofermentative characteristics, except for one heterofermentative sample from the healthy group. Both IBS and healthy groups exhibited strains from *Lactobacillus* and *Enterococcus* genera, with *Enterococcus faecium* being predominant in both. Demographic analysis showed higher IBS prevalence among individuals aged 20–40, with IBS‐C more common in women and IBS‐D in men. The study concluded that individuals with IBS had significantly lower LAB microbiota counts, potentially impacting intestinal defense function. Further exploration of LAB behavioral and immunomodulatory traits may enhance understanding of intestinal microbiota's role in IBS and aid in developing treatment strategies.

## Graphical abstract

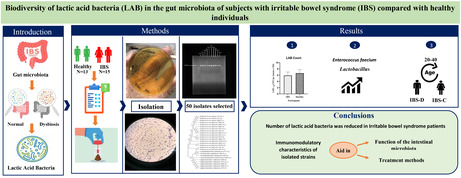

## 1 INTRODUCTION

Irritable bowel syndrome (IBS) is one of the most common functional gastrointestinal disorder (FGID) affecting about 11% of adult population worldwide. Due to the lack of specific and sensitive diagnostic biomarkers, IBS is still diagnosed by symptomatic criteria, that is, Rome IV criteria. It is characterized by abdominal pain and changes in stool consistency and frequency, along with other common symptoms such as abdominal distension and bloating. According to predominant bowel habit, patients are classified into four subgroups: IBS with predominant constipation (IBS‐C), IBS with predominant diarrhea (IBS‐D), mixed IBS (IBS‐M), and IBS without type (IBS‐U) (where the stool pattern cannot accurately include the patient in one of the other three groups).

Although the origin of IBS has remained unresolved, growing evidence suggests that factors including food, bile acids, antibiotics and infections, gender, and psychosocial events are involved. In genetically and epigenetically predisposed individuals, these factors may increase intestinal permeability by causing changes in the function of the intestinal epithelial barrier. As a result, through the activation of local and brain immune responses as well as the neuroendocrine ones and changes in the microbiota, it can cause abnormal secretion and sensory–motor output in the gut, which is related to the duration and severity of the disease symptoms (Rodiño‐Janeiro et al., 2018).

Gut microbiota refers to the microorganisms that reside in various parts of the human body from birth, including skin, oral cavity, vagina, and gastrointestinal tract (GIT). The gastrointestinal tract hosts between 500 and 1000 various species of bacteria, with the exact composition varying from person to person. In adults, the microbiota composition is influenced by factors such as diet, region, and consumption of oral antibiotics. It is also believed that more significant alterations occur in old age. Dysbiosis (also known as dysbacteriosis) refers to microbial imbalance on or in the body and is usually reported in terms of changes in the digestive tract. Many factors can negatively affect the commensal gut microbiota and strengthen dysbiosis, including antibiotic consumption, mental and physical stress, radiation, alteration in peristalsis (intestinal movements), and changes in diet (Moraes‐Filho & Quigley, 2015).

Overall, the data suggest that in IBS, there is a relative increase in proinflammatory bacterial species like *Enterobacteriaceae*, while *Lactobacillus* and *Bifidobacterium* decrease. The ability of *Lactobacillus* and *Bifidobacterium* genera to interact with other bacterial species or the host can lead to modulate the microbiota and immune system (Rodiño‐Janeiro et al., 2018).

LAB are mostly denoted as a nontaxonomic heterogeneous group of Gram‐positive, nonspore‐forming, facultative anaerobic bacteria with low guanine–cytosine (GC) content. They are classified into seven phylogenetic groups, including *Lactobacillus*, *Leuconostoc*, *Enterococcus*, *Lactococcus*, *Pediococcus*, *Streptococcus*, and *Oenococcus*. Accurate identification of LAB is crucial for their technological, ecological, and safety applications (Ali Baradaran et al., 2012).

In recent years, advances in microbial biodiversity and understanding of microbial interactions with the host cells have led to the identification of molecules and systems that can impact health or disease (Pelinescu et al., 2011). LAB and *Bifidobacteria* are the most frequent types of microbes used as probiotics (Zhu et al., 2011). These bacteria are typically found in carbohydrate‐rich media such as plants, fermented foods, and mammalian mucosal surfaces like mouth, gut, and vagina (Dallal et al., 2017). LAB isolated from human gut are particularly advantageous owing to their adaptation to the GIT environment and ability to colonize the gut for long periods. When consumed in adequate amounts, LAB can provide health benefits to the host (Wang et al., 2021).

Traditional physicochemical and biochemical identification methods are often unreliable because of the similar morphological and nutritional requirements of different species. Genotype‐based methods, such as 16S rDNA sequencing, are independent of growth conditions and robust alternatives to the phenotypic ones. The use of 16S rDNA sequencing can be linked to databases that provide up to 100,000 sequences for the phylogenetic framework (Ali Baradaran et al., 2012).

On the other hand, we still lack effective approaches to alter the natural history of the disease. Targeting the microbiome could be one of these approaches, as IBS patients show several qualitative and quantitative changes in fecal microbiota. Therefore, the role of intestinal microbiota appears to be a fundamental feature in creating future therapeutic approaches for IBS (Rodiño‐Janeiro et al., 2018).

This work aimed to isolate indigenous LAB from the feces of people with IBS and healthy people (control), as well as identifying and characterizing their biochemical properties such as resistance to variations in temperature and sodium chloride concentration, acidifying capacity, gas production, Gram staining, and catalase test. Subsequently, a number of bacteria with the best LAB biochemical properties were selected. Finally, alterations in the commensal microbiota were examined using polymerase chain reaction (PCR) and 16S rRNA gene sequencing by comparing the selected LAB isolated from the IBS and healthy individuals under laboratory conditions.

## 2 MATERIALS AND METHODS

### 2.1 Participants

All procedures were performed in accordance with the standard guidelines for the care and use of human subjects and the study conducted at the Department of Food Science and Technology, Faculty of Agriculture, Ferdowsi University of Mashhad and the Department of Laboratory Sciences, School of Paramedical Sciences, Mashhad University of Medical Sciences, both located in Mashhad, Iran.

All the participants were provided with written information, and their written consent to participate was obtained. Stool samples were collected from 15 adults with confirmed IBS and 13 healthy individuals.

The presence of gastrointestinal symptoms, suggestive of IBS according to Rome IV criteria (Lacy & Patel, 2017), was assessed by experienced gastroenterologists and fecal calprotectin level test. In order to obtain the necessary information about the amount and severity of each patient's pain and existing symptoms, a questionnaire was prepared based on the study previously conducted by Kosako et al. (2018). The participants with metabolic and infectious diseases, lactose intolerance, earlier abdominal surgeries, celiac disease, pregnancy, or structural abnormalities of the GIT were excluded. The participants had not taken any probiotics or antibiotics within the 3 months before providing their samples. The mean age of the IBS participants was (mean ± SD) 28.46 ± 6.98 years old, while the adult and infant healthy participants were 52.40 ± 6.18 and 1.66 ± 0.57 years old, respectively, with a male to female ratio of 7:8 in the IBS and 6:7 in the healthy ones (Table 1a).

**TABLE 1:** Characteristics of the (a) participants and (b) 15 irritable bowel syndrome (IBS) patients.

(a)			
Characteristics	IBS group	Healthy group (adult)	Healthy group (infants)
No. of participants	15	10	3
Age (years)	28.46 ± 6.98	52.40 ± 6.18	1.66 ± 0.57
Sex (M:F)	7:8	3:7	3:0

(b)	
IBS characteristics	*n* = 15 (%)
*Subtype*	
Diarrhea	5 (33.33)
Constipation	8 (53.33)
Mixed	2 (13.33)
*Abdominal pain*	
0	2 (13.33)
1	2 (13.33)
2	3 (20.00)
3	7 (46.66)
4	1 (6.66)
*Feeling pain* (*day/week*)	
0 D/W	3 (20.00)
1 D/W	1 (6.66)
2–3 D/W	4 (26.66)
4–5 D/W	4 (26.66)
>5 D/W	3 (20.00)
*Abdominal bloating*	
0	0
1	3 (20.00)
2	6 (40.00)
3	5 (33.33)
4	1 (6.66)
*Bowel movements*	
0	1 (6.66)
1	4 (26.66)
2	7 (46.66)
3	2 (13.33)
4	1 (6.66)

*Note*: Data are mean ± standard deviation.

Results are presented as number (percentage).

0: None, 1: Mild, 2: Moderate, 3: Severe, 4: Critical.

### 2.2 Sampling, isolation, and purification of LAB

Human fecal samples from the intended individuals were collected in sterile containers, kept in an ice box (at 4 ± 1°C), and transported to laboratory within an hour for immediate processing (Gomathi et al., 2014). To isolate LAB, 1 g of each fecal sample was homogenized in 9 mL of 1% peptone water buffered (0.85% NaCl, 0.1% peptone, and 0.01% cysteine at pH 7.0). An enriched culture of 1 mL was serially diluted (10‐fold) in peptone water buffered, and each serial dilution was spread onto De Man, Rogosa, and Sharp (MRS) agar for LAB isolation and MRS supplemented with 0.5% l‐cysteine.HCl for LAB isolation and bifidobacteria growth. The plates were incubated under aerobic and anaerobic conditions at 37°C for 24–48 h using the conventional pour plate or spread method for microbe enumeration (Khalil et al., 2007). The well‐isolated colonies were randomly picked from the MRS plates with 30–300 colonies based on the colony morphology and/or color (Ni et al., 2015). They were then inoculated into MRS broth under the same conditions. The selected colonies were purified by repeated streaking on the corresponding isolation medium plates. Finally, the overnight cultures of all the isolated colonies (Gram‐positive and catalase‐negative) in MRS broth containing 30% glycerol were stored at −80°C as stock cultures. Before being used, the isolated strains were refreshed in MRS broth at 37°C for 24 h under anaerobic conditions and subcultured on MRS agar (Tinrat et al., 2011).

### 2.3 Identification and examination of phenotypic characteristics

#### 2.3.1 Confirmation tests

##### Catalase activity

The overnight cultures of the isolates were grown on MRS agar at 37°C for 24 h under anaerobic conditions. The catalase test was conducted by dripping two drops of hydrogen peroxide (3%) on the 24‐h‐old cultures on a glass slide. The catalase test showed positive reaction characterized by the formation of oxygen bubbles indicating the production of catalase by the tested bacterium. Therefore, the isolates which did not produce gas bubbles were selected for subsequent activities (Kumar et al., 2017).

#### 2.3.2 Morphological, physiological, and biochemical tests of LAB isolates

The LAB isolates were identified according to their morphological, physiological, and biochemical characteristics. In this in vitro study, a basic MRS medium was utilized, and an overnight culture of every individual isolate was employed as an inoculum (Menconi et al., 2014).

##### Cell morphology

The wet mounts of the overnight cultures were prepared on microscopic slides and evaluated under a light microscope with oil immersion objectives. Cellular morphology, including cell shape and arrangements, was taken into consideration during the examination (Mulaw et al., 2019).

##### Acidifying capacity

To test the acidification capacity, vials containing 10 mL of MRS broth (pH 7.0) were used. After incubation at 32°C for 24 h, the pH value was determined using an AZ86502 pH meter (AZ instruments) (Greco et al., 2005).

##### Gas production from LAB isolates

In order to determine the homofermentative and heterofermentative characteristics of the LAB isolates, CO_2_ production from glucose was measured in modified MRS broth containing 1% glucose with inverted Durham tubes. The culture medium was prepared in a glass test tube with Durham tubes immersed in the broth. An overnight culture of the LAB isolates (5% v/v) was inoculated into the broth and covered with sterile oil to prevent oxidation before being incubated at 30°C for 48 h under anaerobic conditions. If CO_2_ was produced from the fermented glucose, the result was declared positive (Ali Baradaran et al., 2012).

##### Growth at different temperatures

Tubes containing MRS broth were inoculated with the pure colonies grown on MRS agar and incubated in anaerobic jars at different temperatures (15°C, 30°C, 37°C, and 45°C) for 72 h. If turbidity was observed after 72 h, positive results would be reported (Dallal et al., 2017).

##### Growth at different NaCl concentrations

To assess the resistance of the LAB isolates to varying NaCl concentrations, 4% and 6.5% (w/v) NaCl concentrations were selected for testing. Test tubes containing 5 mL of MRS broth and the respective NaCl concentrations were prepared and inoculated with 50 μL of 1% overnight culture of each LAB isolate. Next, the tubes were incubated at 37°C for 7 days, and growth was observed visually by the appearance of turbidity (Mulaw et al., 2019).

##### Growth at different pH values

The pH resistance test was performed to select the LAB isolates which could still grow in MRS broth with acidic environment at pH values of 3, 4, and in neutral conditions, that is, pH 7. For this purpose, 5‐mL tubes of acidified MRS broth were inoculated with 1% overnight culture of the LAB isolates and further incubated at 30°C for 7 days. Growth was marked by turbidity in the media (Ni et al., 2015).

### 2.4 Genotypic characterization

#### 2.4.1 Genomic DNA preparation for PCR and sequencing

The overnight culture of each strain was streak plated on MRS agar (Sigma‐Aldrich) and incubated at 37°C under anaerobic conditions for 48 h. Genomic DNA was extracted from a single colony of each strain using the SinaPure™ DNA extraction kit (Sinaclon) based on the manufacturer's instructions. The DNA concentration was determined using a microplate reader (Biotek‐Epoch7) at 260 nm, and its quality was assessed by the ratio of the absorbance readings at 260 and 280 nm. Furthermore, electrophoresis was performed on 1% agarose gel in TBE 1X buffer and photographed under UV light. DNA was stored at −20°C and used for all the PCRs mentioned in this study (Adimpong et al., 2012).

#### 2.4.2 16S rRNA Gene sequencing

All the isolated strains were identified by genetic analysis through PCR and 16S rRNA sequencing. The universal PCR primers 27F (5′‐AGAGTTTGATCCTGGCTCAG‐3′) and 1492R (5′‐GGTTACCTTGTTACGACTT‐3′) (Macrogen) were employed to amplify the 16S rRNA gene (Wang et al., 2021). PCR was carried out in a thermocycler (Sens Quest‐labcycler) in a total volume of 25 μL containing 12.5 μL 2X PCR master mix (SinaClon), 1 μL of each of the forward and reverse primers (10 pmol/μL), 6.5 μL of sterile nuclease‐free deionized water, and 4 μL of template DNA, running under the following temperature program: initial denaturation of DNA at 95°C for 5 min, 35 amplification cycles of 30‐s denaturation at 94°C, annealing at 55°C for 45 sec, plus 2‐min elongation at 72°C, and a final 10‐min extension step at 72°C. The tubes were cooled to 4°C (Emerenini et al., 2013). Five‐microliter aliquots of the PCR products in combination with 1 μL of loading buffer were analyzed by electrophoresis using a 1% (w/v) agarose gel in Tris boric acid EDTA (TBE 1X) buffer, stained with ethidium bromide (0.5 μg mL^−1^), at 75 V for 75–90 min. Afterward, the gel was placed in Gel doc (Biomedical sab2‐image pad M1) to detect the presence of a band of 1500 bp. The size of the DNA fragments was estimated using a GeneRuler 100 bp Plus DNA Ladder (SinaClon). The PCR products were sent to Pishgam Laboratories for sequencing. Single‐pass sequencing was conducted on each template using primer 1492R.

#### 2.4.3 Phylogenetic analysis

An average of 1000 bp nucleotides were read for each sequence from one side, and the sequence homologies were examined by comparing the obtained sequences with the 16S rRNA ones available in the National Center of Biotechnology Information (NCBI) GenBank gene database website (http://blast.ncbi.nlm.nih.gov/Blast.cgi) using the Basic Local Alignment Search Tool (BLAST) program (Lacy & Patel, 2017). The isolates with a minimum of 98% similarity in sequences were considered the same species. Multiple sequence alignments were analyzed using BioEdit software version 7.0.0. To construct a phylogenetic tree and to compare similarities among the sequences by the neighbor‐joining method, MEGA software version 11 was applied (https://www.megasoftware.net/) (Tamura et al., 2021). The stability of the tree was evaluated by bootstrap method using 1000 replications (Sulistiani et al., 2014).

### 2.5 Statistical analysis

The data were analyzed using descriptive statistics, mean, standard deviation, and graphs. Microbial counts were expressed as log10 colony‐forming units (CFU) per gram feces, and the results were presented as mean ± standard deviation. Statistical analysis was performed using GraphPad Prism 9.5.1, while the correlation diagrams were generated using Python software version 3.8. BioEdit and Mega11 were utilized for sequence editing and phylogenetic tree design, respectively.

## 3 RESULTS

### 3.1 IBS diagnosis and symptoms

Irritable bowel syndrome (IBS) was diagnosed by a gastroenterologist and differentiated by calprotectin test from healthy people and those with inflammatory bowel disease (IBD). The results showed that calprotectin levels in individuals with IBS were higher than in healthy individuals, but lower than in those with IBD. The IBS symptom scores and subtypes are shown in Table 1b according to the questionnaire answered by the participants. The individuals with IBS‐C constituted almost half of the patients' population, while the ones with IBS‐D and IBS‐M, respectively, accounted for 33.33% and 13.33% of the studied patients. Of the ones with IBS, 46.66% experienced severe pain lasting for 2–5 days a week in almost half of the population. Abdominal bloating and bowel movements were also reported to be moderate in most of the participants.

### 3.2 Enumeration of isolates and phenotypic properties

**FIGURE 1:**
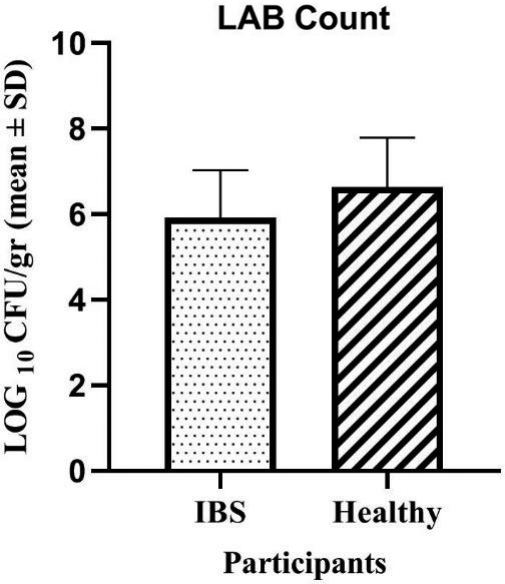
The number (mean ± standard deviation) of lactic acid bacteria (LAB) in the feces of IBS‐suffering and healthy individuals.

The viable counts of the LAB present in the samples are summarized in Table 2. The total counts of the LAB varied from 4.07 to 7.78 and 5.23 to 9.84 log CFU mL^−1^ in 15 IBS and 13 healthy fecal samples, respectively. The average LAB counts in the samples of the IBS and healthy individuals were 5.91 and 6.63 log CFU mL^−1^, respectively (Figure 1). The Gram‐positive and catalase‐negative bacteria growing on MRS agar were considered presumptive LAB. In total, 50 presumptive LAB were selected from 82 isolates given their Gram staining and catalase activity.

**TABLE 2:** Viable cell count of lactic acid bacteria (LAB) in stool.

Participant's code (healthy)	LAB count	Participant's code (IBS)	LAB count
MH.H	8.678766618	ME.S	4.072550667
MA.H	7.735308499	ME.S.L	4.072550667
AG.H	5.354806662	PG.S	5.119975317
ME.H	5.827839035	PG.S.L	4.674610658
ME.H.L	5.943134628	MH.S	7.592580471
SM.H	6.401870302	MH.S.L	7.78142896
AG.H.L	5.447862483	AM.S.L	6.534948665
SM.H.L	7.913332106	SM.S.L	4.685334524
FA.H	7.768503561	JV.S.L	5.861697302
FA.H.L	6.649688807	JV.S	5.915255894
FG.H	5.231608587	MJ.S.L	7.168122329
FG.H.L	5.736758565	MJ.S	6.746775686
MA.H.L	5.402652111	ZP.S	5.861697302
AV.H	6.41039375	ZP.S.L	6.847909017
FF.H	6.520900179	MA.S	6.409625767
FF.H.L	6.722035308	MA.S.L	5.28082661
AV.H.L	5.96933118	BA.S	5.59207577
FN.H.L	7.429899026	BA.S.L	5.677109004
MJ.H.L	6.729459326	FF.S	5.806796432
FN.H	7.335184272	FF.S.L	6.144715695
MJ.H	6.648803395	PGH.S.L	7.520900179
BA.H	9.841416356	PGH.S	7.581856605
NG.H.L	5.934039123	MEJ.S.L	6.052029
NG.H	5.702508865	MEJ.S	5.994037053
		FS.S	4.455536963
		FS.S.L	4.450668919

*Note*: Unit: Log_10_ colony‐forming unit (CFU)/g sample.

On MRS agar, the strains of both the IBS and healthy groups produced round milky white colonies of 1–2 mm in diameter, with regular edges, a slightly raised center, and an opaque smooth surface. The majority of the isolated strains were cocci (65.38% in the IBS participants and 75% in the healthy ones), and the remainders were rod shaped (34.61% in the IBS group and 25% in the healthy one). The cells of the isolates in the coccus type were arranged either in pairs or single, while the rod‐shaped cells were almost single, except the MA.H.L one (Table 3a,b). The morphology of the two strain groups was assessed using scanning electron microscopy.

**TABLE 3:** Physiological, morphological, and biochemical properties as well as comparative growth of the lactic acid bacteria (LAB) isolates upon exposure to different conditions of pH, temperature, and salt (NaCl) concentration in (a) irritable bowel syndrome (IBS) and (b) healthy individuals.

Isolates	Cell shape	Cell arrangement	Gram staining	Catalase	Production		Growth at temperature (°C)				Growth in NaCl (%)		Growth in pH		
					Gas	Acid	15	30	37	45	4	6.5	3	4	7
**(a)**															
ME.S	Cocci	Single	+	−	−	+	+	+	+	+	+	+	+	+	+
ME.S.L	Cocci	Single	+	−	−	+	+	+	+	+	+	+	+	+	+
PG.S	Cocci	Single, Pairs	+	−	−	+	+	+	+	+	+	+	+	+	+
PG.S.L	Cocci	Single	+	−	−	+	W	+	+	+	+	+	+	+	+
MH.S	Cocci	Single	+	−	−	+	+	+	+	−	+	+	+	+	+
MH.S.L	Cocci	Single	+	−	−	+	+	+	+	−	+	+	+	+	+
AM.S.L	Cocci	Single	+	−	−	+	W	+	+	+	+	+	+	+	+
SM.S.L	Rod	Single	+	−	−	+	+	+	+	+	+	−	W	+	+
JV.S.L	Cocci	Single	+	−	−	+	+	+	+	−	W	−	−	+	+
JV.S	Cocci	Single	+	−	−	+	+	+	+	−	+	−	+	+	+
MJ.S.L	Cocci	Single	+	−	−	+	+	+	+	+	+	W	+	+	+
MJ.S	Rod	Single	+	−	−	+	+	+	+	+	+	+	−	+	+
ZP.S	Cocci	Single	+	−	−	+	+	+	+	+	+	W	+	+	+
ZP.S.L	Cocci	Pairs, Chains	+	−	−	+	+	+	+	+	+	W	+	+	+
MA.S	Rod	Single	+	−	−	+	−	+	+	+	+	+	+	+	+
MA.S.L	Rod	Single	+	−	−	+	+	+	+	+	+	+	+	+	+
BA.S	Rod	Single	+	−	−	+	+	+	+	+	W	W	−	+	+
BA.S.L	Rod	Single	+	−	−	+	+	+	+	+	W	−	W	W	+
FF.S	Cocci	Pairs, Chains	+	−	−	+	+	+	+	+	+	+	+	+	+
FF.S.L	Rod	Single	+	−	−	+	+	+	+	+	+	+	+	+	+
PGH.S.L	Cocci	Pairs, Chains	+	−	−	+	−	+	+	+	+	W	+	+	+
PGH.S	Cocci	Pairs, Chains	+	−	−	+	−	+	+	+	W	−	W	+	+
MEJ.S.L	Cocci	Single	+	−	−	W	−	+	+	+	+	W	−	+	+
MEJ.S	Cocci	Single	+	−	−	+	+	+	+	+	+	W	+	+	+
FS.S	Rod	Single	+	−	−	+	+	+	+	+	+	W	+	+	+
FS.S.L	Rod	Single	+	−	−	+	+	+	+	+	+	+	+	+	+
**(b)**															
MH.H	Cocci	Single	+	−	−	+	−	+	+	−	+	+	W	+	+
MA.H	Cocci	Single	+	−	−	+	+	+	+	+	+	+	+	+	+
AG.H	Cocci	Pairs, Chains	+	−	−	+	+	+	+	+	+	+	+	+	+
ME.H	Cocci	Single	+	−	−	+	+	+	+	+	+	+	W	+	+
ME.H.L	Cocci	Single	+	−	−	+	+	+	+	+	+	+	+	+	+
SM.H	Cocci	Single	+	−	−	+	+	+	+	+	+	+	+	+	+
AG.H.L	Cocci	Pairs, Chains	+	−	−	+	+	+	+	+	+	+	+	+	+
SM.H.L	Cocci	Single	+	−	−	+	W	+	+	+	+	+	−	+	+
FA.H	Rod	Single	+	−	−	+	+	+	+	+	+	+	+	+	+
FA.H.L	Rod	Single	+	−	+	+	+	+	+	+	+	+	+	+	+
FG.H	Rod	Single	+	−	−	+	+	+	+	+	+	W	+	+	+
FG.H.L	Rod	Single	+	−	−	+	+	+	+	+	+	+	+	+	+
MA.H.L	Rod	Single, Chains	+	−	−	+	W	+	+	+	+	+	+	+	+
AV.H	Cocci	Single	+	−	−	+	+	+	+	−	W	−	W	+	+
FF.H	Cocci	Single	+	−	−	+	+	+	+	+	+	W	+	+	+
FF.H.L	Cocci	Single	+	−	−	+	+	+	+	−	W	−	+	+	+
AV.H.L	Rod	Single	+	−	−	W	+	+	+	−	W	W	−	+	+
FN.H.L	Cocci	Pairs, Chains	+	−	−	+	+	+	+	+	W	−	W	+	+
MJ.H.L	Cocci	Single	+	−	−	+	+	+	+	+	W	−	+	+	+
FN.H	Cocci	Single	+	−	−	+	+	+	+	+	W	−	+	+	+
MJ.H	Cocci	Pairs	+	−	−	+	+	+	+	+	+	W	+	+	+
BA.H	Cocci	Single	+	−	−	+	+	+	+	+	+	W	+	+	+
NG.H.L	Cocci	Pairs, Chains	+	−	−	+	+	+	+	+	+	+	+	+	+
NG.H	Cocci	Pairs, Chains	+	−	−	W	+	+	+	+	+	+	+	+	+

*Note*: Growth criteria: +, positive; w, weakly positive; −, negative.

Abbreviations: The first letter of each sample, the first letter of each sample's first name; the second letter of each sample, the first letter of each sample's second name; S, the first letter of sick; H, the first letter of healthy; L, use of l‐cysteine. HCl in the culture for growing of isolated bacteria.

### 3.3 Biochemical and physiological characterization

The results of the biochemical and physiological measurements of the isolates (Table 3a,b) indicated that all the 50 Gram‐positive and catalase‐negative isolates had the ability to produce lactic acid but not CO_2_ from glucose, except for the FA.H.L strain from the group of healthy people, which showed the presence of gas in the Durham tube.

Regarding the resistance characteristics (Table 3a,b), all the isolates recorded maximum growth at 37°C and 30°C with comparatively weaker growth at 15°C for two isolates of both the IBS and healthy groups. However, four isolates of the IBS and one isolate of the healthy groups did not grow at 15°C. In addition, four isolates of both the IBS and healthy individuals exhibited no growth at 45°C. Likewise, there were many variations among the tested isolates when grown in media with low (4%) or high (6.5%) NaCl concentration such that weak growth was observed for four isolates of the IBS and six isolates of the healthy groups at low NaCl concentration and for eight isolates of the IBS and five isolates of the healthy groups at high NaCl concentration. At 6.5% NaCl, five isolates grew from neither the IBS nor the healthy groups. The pH resistance test revealed that 22 (84.61%) isolates of the IBS and 22 (91.66%) isolates of the healthy groups could grow at pH 4.5, of which three isolates from the IBS group and four isolates from healthy one had weak growth, whereas 4 (15.38%) isolates of the IBS individuals and 2 (8.33%) isolates of the healthies could not grow at that pH. At the same time, at higher pH values (4 and 7), all of the isolates grew well except BA.S.L from the IBS group, which showed weak growth.

### 3.4 Genotypic characterization

#### 3.4.1 DNA genome of LAB

**FIGURE 2:**
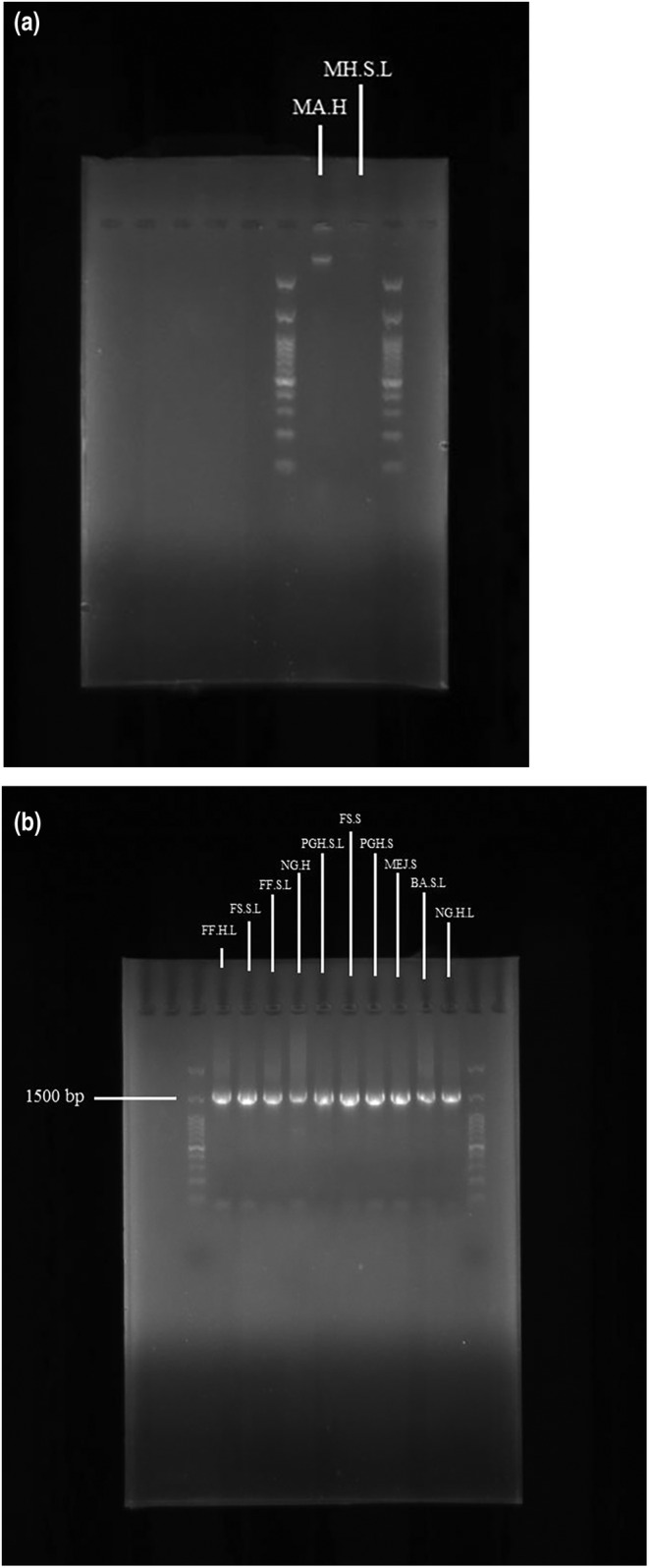
DNA band formation (a) in agarose gel and (b) PCR product bands on the marker at ~1500 bp, using 27F and 1492R primers.

The DNA band formation in agarose gels showed the migration pathway of the DNA with varying band thicknesses (Figure 2a). As a result of measuring the absorbance ratio of 260–280 nm in the microplate reader, all the samples showed a good quality with a ratio between 1.7 and 2.

#### 3.4.2 16S rRNA gene of LAB

Based on the amplification results, the DNA bands of the isolates are presented in Figure 2b demonstrating parallel DNA bands on the marker at ~1500 bp. Using 27F and 1492R primers, all the 50 LAB isolates had successfully amplified 16S rRNA genes with expected DNA fragment sizes of ~1500 bp.

#### 3.4.3 16S rRNA sequences and phylogenetic analysis

**FIGURE 3:**
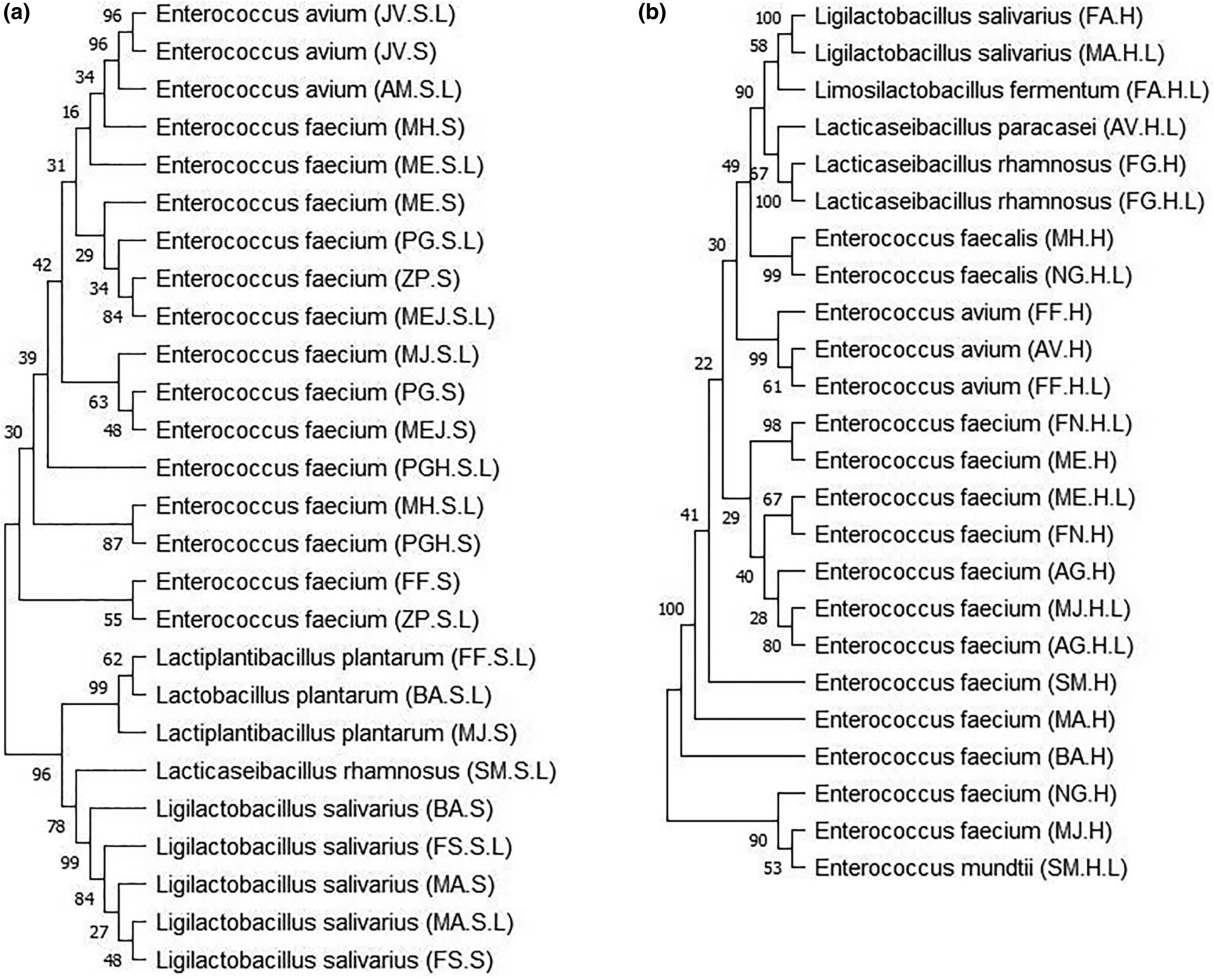
Phylogenetic analysis of the representative strains of the (a) irritable bowel syndrome (IBS) and (b) healthy group.

The PCR products of the 50 strains were sequenced. After that, their 16S rRNA gene sequences were deposited in GeneBank and assigned to the accession numbers indicated in Table 4. The species were initially determined by the BLAST program on NCBI with more than 95% similarity for all the isolates except for seven of them, which showed a similarity percentage above 90% (Table 4). Next, the phylogenetic analysis of the representative strains of each IBS and healthy group was performed to reveal the species and distribution of the various LAB. The isolated strains were used to construct the phylogenetic trees (Figure 3a,b).

**TABLE 4:** Identities of lactic acid bacteria (LAB) isolates through sequencing and GeneBank.

Sample	Scientific name	Identity (%)	Gene bank accession no.	Sample	Scientific name	Identity (%)	Gene bank accession no.
MJ.S.L	*Enterococcus faecium*	98.80	MN833007.1	FN.H.L	*Enterococcus faecium*	99.67	CP118955.1
MH.S.L	*Enterococcus faecium*	96.68	MT545043.1	MA.H	*Enterococcus faecium*	98.95	MT378135.1
ZP.S	*Enterococcus faecium*	99.35	MH976720.1	AG.H	*Enterococcus faecium*	99.89	KR265158.1
MA.S	*Ligilactobacillus salivarius*	100	CP123971.1	AV.H	*Enterococcus avium*	99.89	AP019814.1
AM.S.L	*Enterococcus avium*	99.89	AP019814.1	FA.H	*Ligilactobacillus salivarius*	99.78	CP123971.1
JV.S.L	*Enterococcus avium*	99.89	AP019814.1	FA.H.L	*Limosilactobacillus fermentum*	99.89	CP124737.1
MA.S.L	*Ligilactobacillus salivarius*	99.35	CP123971.1	FG.H	*Lacticaseibacillus rhamnosus*	97.45	MT611887.1
MEJ.S.L	*Enterococcus faecium*	99.44	AP027294.1	FG.H.L	*Lacticaseibacillus rhamnosus*	97.45	OQ346358.1
ME.S	*Enterococcus faecium*	100	CP118955.1	ME.H	*Enterococcus faecium*	99.67	CP118548.1
ME.S.L	*Enterococcus faecium*	99.56	CP118955.1	ME.H.L	*Enterococcus faecium*	99.35	CP118548.1
PGH.S.L	*Enterococcus faecium*	98.91	MT545041.1	MJ.H.L	*Enterococcus faecium*	99.56	CP118955.1
PG.S	*Enterococcus faecium*	99.35	MT378124.1	SM.H	*Enterococcus faecium*	99.46	CP118548.1
PG.S.L	*Enterococcus faecium*	100	KR265158.1	AG.H.L	*Enterococcus faecium*	100	CP118955.1
SM.S.L	*Lacticaseibacillus rhamnosus*	99.89	MT613492.1	FF.H	*Enterococcus avium*	99.78	AP019814.1
BA.S	*Ligilactobacillus salivarius*	97.18	CP123986.1	FF.H.L	*Enterococcus avium*	100	AP019814.1
FF.S	*Enterococcus faecium*	91.91	KX364714.1	FN.H	*Enterococcus faecium*	99.56	CP118955.1
FF.S.L	*Lactiplantibacillus plantarum*	97.93	MF424544.1	MH.H	*Enterococcus faecalis*	99.34	CP124778.1
FS.S	*Ligilactobacillus salivarius*	99.23	CP123971.1	MJ.H	*Enterococcus faecium*	91.53	MF369932.1
FS.S.L	*Ligilactobacillus salivarius*	99.89	CP123971.1	NG.H.L	*Enterococcus faecalis*	99.34	CP124778.1
JV.S	*Enterococcus avium*	99.89	AP019814.1	BA.H	*Enterococcus faecium*	93.66	MT597571.1
MEJ.S	*Enterococcus faecium*	99.56	CP064279.1	MA.H.L	*Ligilactobacillus salivarius*	99.45	CP123971.1
MH.S	*Enterococcus faecium*	99.89	CP066205.1	NG.H	*Enterococcus faecium*	91.83	KX267889.1
PGH.S	*Enterococcus faecium*	95.10	MT597553.1	SM.H.L	*Enterococcus mundtii*	91.56	MW135234.1
BA.S.L	*Lactobacillus plantarum*	91.99	JQ301796.1	AV.H.L	*Lacticaseibacillus paracasei*	91.98	OL469074.1
MJ.S	*Lactiplantibacillus plantarum*	98.05	OQ193027.1				
ZP.S.L	*Enterococcus faecium*	97.03	MT378113.1				

In the IBS group, 26 isolates were categorized into five species and subspecies. *Enterococcus faecium* included 14 strains occupying 53.84% of the entire isolates. *Ligilactobacillus* subsp. *salivarius* had five isolates, which was the second big group with 19.23%. Three isolates of *Lactiplantibacillus plantarum* and *Enterococcus avium* were the third group with about 11.53%. The lowest percentage of the isolated strains was related to *Lacticaseibacillus rhamnosus* which accounted for 3.84%. In the healthy group, 24 isolates were classified into seven species and subspecies. Among them, half of the isolates were identified as *E. faecium*. *Enterococcus avium* was the second group with three isolates (12.5%). Two isolates of each *Enterococcus faecalis*, *Li. salivarius*, and *La. rhamnosus* belonged to the third group with about 8.33%, and the lowest percentage (4.16%) was associated with *Limosilactobacillus fermentum*, *Lacticaseibacillus paracasei*, and *Enterococcus mundtii* with one isolate for each.

From the results before, it can be concluded that *E. faecium* was the predominant LAB species present in the feces of both the IBS and healthy participants.

### 3.5 Correlation of variables

**FIGURE 4:**
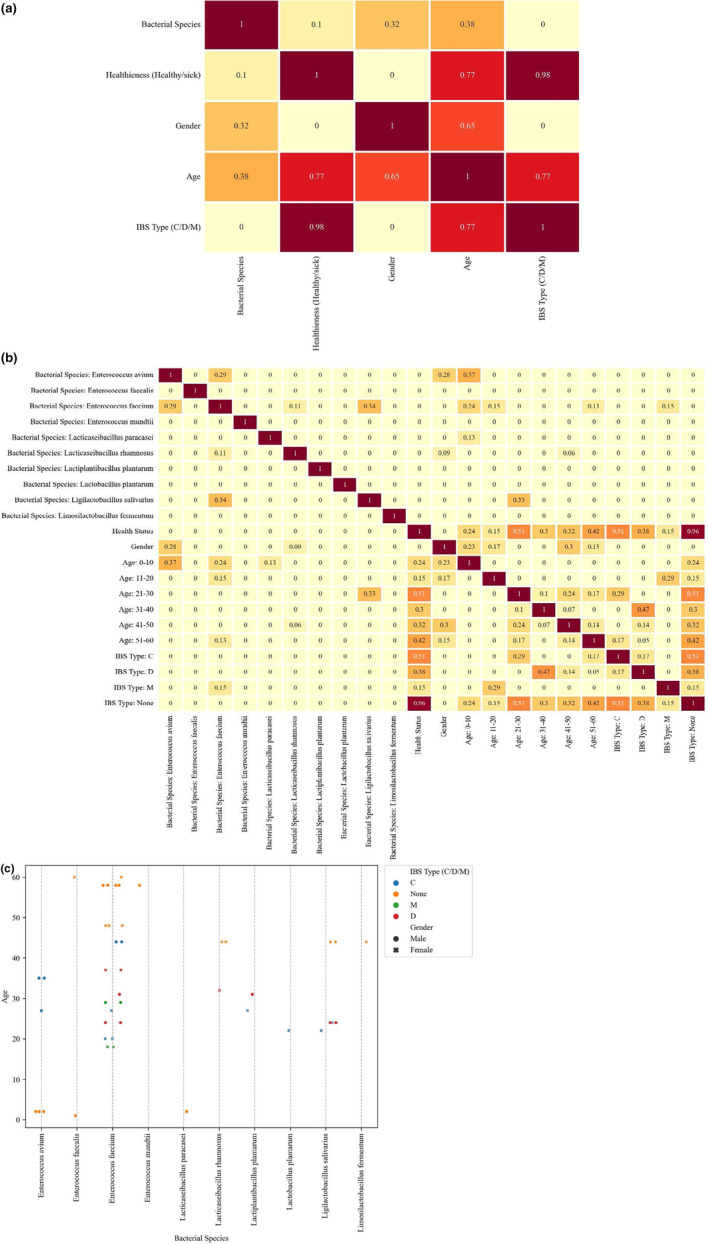
(a) General and (b) detailed correlation, as well as (c) the relationships between all the variables including the identified bacterial strains, the health status of the community [whether they are healthy or irritable bowel syndrome (IBS) suffering], the type of IBS if present, the age and gender of the participants.

This study focused on several variables including the identified bacterial strains, the health status of the community (whether they are healthy or IBS suffering), the type of IBS if present, as well as the age and gender of the participants. The correlation between these variables is depicted in Figure 4a,b in detail.

Notably, the age group of 21–30 had the highest correlation with the IBS‐None and health status, as most of the samples were in this age group which was free of healthy samples. Moreover, over 50% of this group (7 of 13 samples) had IBS‐C, making it highly correlated with this IBS type compared with IBS‐D and IBS‐M. Similar correlations were observed for the other age groups and IBS types such as:

1. IBS‐None and age group 0–10 (100% of the samples).
2. IBS‐D and age group 31–40 (71.5% of the samples).
3. IBS‐None (healthy) and age group 51–60 (83.3% of the samples).

Compared with the other age groups, 11–20 years old demonstrated the lowest correlation with health status and IBS‐None, because there were only four samples in this category, none of which had IBS‐None.

Considering that 50% of the patients had IBS‐C, this group had the highest correlation with health status and IBS‐None. IBS‐D, with a share of about 35% of the patient population, ranked second in terms of correlation with health status and IBS‐None.

Given that all the six samples identified as *E. avium* were isolated from the male participants, three of which were in the age group of 0–10, there was a correlation between this species and the participants' gender as well as the age group. This conclusion is also true for *La. rhamnosus* species and the female gender.

*Enterococcus faecium* had a high frequency; nevertheless, symmetrical gender distribution reduced its correlation with gender. In addition, considering that five of seven strains of *Lactobacillus salivarius* were isolated from the age group of 21–30, there was a strong correlation between this species and the age group. IBS‐M patients all had *Enterococcus faecium* species, indicating a correlation between this species and IBS‐M.

The relationships between all the variables are illustrated in Figure 4c using the swarm plot diagram.

## 4 DISCUSSION

Gut microbiota is crucial for the preservation and performance of this ecosystem. Dysbiosis has been linked to IBD like Crohn's disease and ulcerative colitis. There is growing evidence suggesting that IBS has an inflammatory basis, making it necessary to investigate microbial dysbiosis in IBS (Codling et al., 2010).

The composition of gut microbiota in individuals with IBS compared to healthy individuals has garnered significant attention in recent research (Codling et al., 2010; Kassinen et al., 2007; Noor et al., 2010). While these investigations have successfully highlighted various differences between these populations, there remains a notable gap in the specific examination of lactic acid bacteria (LAB) within the gut microbiota. Most existing studies have concentrated on the broader role of microbiota in IBS and healthy individuals (Chassard et al., 2012; Tap et al., 2017), or have explored therapeutic interventions such as probiotics for managing IBS symptoms (Ford et al., 2018; Harris & Baffy, 2017; Herndon et al., 2020). Consequently, the biodiversity of LAB has not been thoroughly investigated, leaving a critical area of exploration underrepresented. This study aims to fill that void by examining the biodiversity of intestinal LAB microbiota in both healthy and IBS‐affected subjects. Additionally, it seeks to elucidate the relationship between this microbiota and other influencing factors, such as age and gender, through the isolation and identification of these microorganisms using advanced biochemical and molecular techniques.

As reported by Ballou and Keefer (2017) and Fadgyas‐Stanculete et al. (2014), IBS pain can significantly impact daily life. In this research, based on patient questionnaires, 46.66% of the patients experienced pain for 5–6 days a week, which can affect various aspects of life, including work, housework, and social activities (Ballou & Keefer, 2017).

Probiotics and antibiotics can alter the intestinal microbiota (Ford et al., 2018; Harris & Baffy, 2017; Herndon et al., 2020). To prevent probiotic medicine or foods from interfering with our target bacteria, we excluded patients from our study, who had been consuming these items based on their questionnaire responses.

Camilleri (2021) and Codling et al. (2010) evaluated intestinal microbiota through feces and mucus with the aim of obtaining its diversity and composition in the guts of IBS patients. However, to avoid possible damage to the microbiota, we accessed the gut microbiota through feces, in addition to the fact that this is a more accessible method (Tap et al., 2017), similar to Kassinen et al. (2007).

Our stool culture results revealed that the population of LAB in the IBS patients is different from healthy individuals, with an average number of 5.91 in the sick people and 6.63 in the healthy ones (*p* = .029). Chassard et al. (2012) also concluded that the number of important LAB, *Lactobacillus* and *Enterococcus*, was higher in healthy people than in sick ones (*p* = .0175).

Furthermore, MRS and MRS + l‐cysteine.HCl culture media were employed for the growth of the bacteria to investigate the aerobic and anaerobic populations of the LAB. The ability of LAB to grow in both aerobic and anaerobic conditions can suggest the facultative anaerobic nature of some LAB strains such as *Enterococci* and *Lactobacilli*. On the other hand, our study found no significant difference in the number of facultative anaerobic LAB between the healthy and IBS groups, which is consistent with the results reported by Mättö et al. (2005).

One of the characteristics of LAB is their glucose consumption method. The first group produces only lactic acid by consuming glucose (homofermentative), but the second one produces gas in addition to lactic acid (heterofermentative) (Wood, 1992). According to the research conducted by Wang et al. (2021) on LAB isolated from infant feces, 16 bacteria were isolated, all of which were identified as homofermentative. In this study, except for one isolate from the group of healthy people, none of the LAB isolates were gas producing. Based on Mikelsaar et al. (2016) studies on the biodiversity of intestinal LAB in a healthy population, LAB can be a group of facultative heterofermentatives, including *L. casei*, *L. paracasei*, *L. plantarum*, and *L. rhamnosus*, which do not produce gas after glucose consumption.

Recent research has revealed that individuals with IBS have a distinct gut microbiota, compared with healthy people (Camilleri, 2021; Carroll et al., 2011; Codling et al., 2010). Traditional culture‐based techniques, which lack complete precision and accuracy (Liu et al., 2017), are not effective enough in studying the intestinal microbiota as they are resistant to these methods (Codling et al., 2010). Therefore, we employed molecular methods to identify the isolates.

*Bacteroidetes* and *Firmicutes* are the dominant populations of intestinal microbiota (Herndon et al., 2020; Zhuang et al., 2018). Since the population in this research had the LAB belonging to the order of *Firmicutes*, and *Enterococcus* is also classified as LAB (Mikelsaar et al., 2016; Růžičková et al., 2020), the species of this genus accounted for the largest number in both the healthy and IBS‐suffering individuals using the identification method of 16S rRNA gene sequencing. However, the order *Firmicutes* was less prevalent in the IBS patients compared with the healthy individuals. These findings are aligned with the studies carried out by Zhuang et al. (2018) who examined the microbial population in stool samples associated with IBS‐D.

Camilleri (2021) demonstrated that microbial population in IBS patients differs in terms of diversity, compared with healthy individuals, and that the microbial diversity decreases with a change in health status from healthy individuals to IBS patients and further to individuals with acute IBD. In contrast, our study did not find a significant difference in microbial diversity between the sick and healthy individuals. The only difference observed was in their resistance to harsh conditions such as pH, temperature, and salt, which can be attributed to the type of strain, that is, specific strain of LAB used (Ljungh & Wadstrom, 2006).

Studies performed by Fadgyas‐Stanculete et al. (2014), Kim and Kim (2018), and El‐Salhy et al. (2014) suggest that IBS is more prevalent in women, particularly those under 50 years old. Gender‐related differences in IBS incidence become apparent around puberty, with women experiencing a higher incidence than men. However, as individuals age, the incidence of IBS decreases in women but remains constant in men between the ages of 20 and 70. Our research found out that IBS was most prevalent among individuals aged 20–40, with no significant difference observed between genders. This lack of gender difference may be attributed to various factors including clinical environment in terms of race, geography, the number of men and women available to participate, or data collection methods (Kim & Kim, 2018).

Regarding the subgroups of IBS, it has been realized that IBS with predominant constipation is more common in women compared with men. In a recent study, it was observed that women took up 30.76% of the IBS‐C patient population, while only 19.23% were men. This difference in prevalence could potentially be influenced by sex hormones and their associated levels of stress experienced by individuals (Kim & Kim, 2018).

## 5 CONCLUSIONS

To summarize, our sequencing results found no significant difference in LAB strain diversity between healthy and IBS‐suffering individuals, with *E. faecium* being the most prevalent strain in both groups. However, resistance characteristics varied depending on the strain and environmental conditions, with healthy stool isolates exhibiting higher resistance. Gender and age were also found to impact IBS, with a higher prevalence among individuals aged 20–40 and IBS‐C more common in women, while IBS‐D in men. Understanding these differences can lead to improved clinical outcomes. Further investigation into fecal LAB microbiota in terms of probiotic and immunomodulatory properties in IBS patients compared with healthy controls is necessary to develop optimal personalized therapeutic strategies and clarify the underlying pathophysiology of IBS. Overall, our study suggests that a reduction in the number of LAB in IBS patients may indicate a loss of protective function rather than a specific organism contributing to the development of IBS.

## AUTHOR CONTRIBUTIONS

**Aysooda Azimi:** Conceptualization (equal); data curation (equal); formal analysis (equal); investigation (equal); methodology (equal); resources (equal); writing – original draft (equal); writing – review and editing (equal). **Masoud Yavarmanesh:** Conceptualization (equal); methodology (equal); project administration (equal); supervision (equal); writing – review and editing (equal). **Mehran Gholamin:** Methodology (equal); project administration (equal); supervision (equal).

## FUNDING INFORMATION

This study was supported by a grant (no. 55005) from the research deputy of the Ferdowsi University of Mashhad, Iran.

## CONFLICT OF INTEREST STATEMENT

The authors declare that they have no conflict of interest and that they have no actual or potential competing financial interests.

## ETHICS STATEMENT

This study involves questionnaire, fecal and blood samples testing from human subjects. All applicable international, national, and/or institutional guidelines for the care and use of human subjects were followed, and a written informed consent was obtained from every patient before the study. The ethical criteria with the code of IR.UM.REC.1400.041 (Ferdowsi University of Mashhad, Iran) were observed.

## Data Availability

The data generated or analyzed that support the findings of this study are available and included in this published article.

## References

[fsn34477-bib-0001] Adimpong, D. B. , Nielsen, D. S. , Sørensen, K. I. , Derkx, P. M. , & Jespersen, L. (2012). Genotypic characterization and safety assessment of lactic acid bacteria from indigenous African fermented food products. BMC Microbiology, 12(1), 1–12. 22594449 10.1186/1471-2180-12-75 PMC3463448

[fsn34477-bib-0002] Ali Baradaran, A. B. , Foo HooiLing, F. H. , Sieo ChinChin, S. C. , & Raha Abdul Rahim, R. A. R. (2012). Isolation, identification and characterization of lactic acid bacteria from *Polygonum minus* .

[fsn34477-bib-0003] Ballou, S. , & Keefer, L. (2017). The impact of irritable bowel syndrome on daily functioning: Characterizing and understanding daily consequences of IBS. Neurogastroenterology and Motility, 29(4), e12982. 10.1111/nmo.12982 PMC5367953 27781332

[fsn34477-bib-0004] Camilleri, M. (2021). Diagnosis and treatment of irritable bowel syndrome: A review. JAMA, 325(9), 865–877. 33651094 10.1001/jama.2020.22532

[fsn34477-bib-0005] Carroll, I. M. , Ringel‐Kulka, T. , Keku, T. O. , Chang, Y.‐H. , Packey, C. D. , Sartor, R. B. , & Ringel, Y. (2011). Molecular analysis of the luminal‐and mucosal‐associated intestinal microbiota in diarrhea‐predominant irritable bowel syndrome. American Journal of Physiology. Gastrointestinal and Liver Physiology, 301(5), G799–G807. 21737778 10.1152/ajpgi.00154.2011 PMC3220325

[fsn34477-bib-0006] Chassard, C. , Dapoigny, M. , Scott, K. P. , Crouzet, L. , Del'Homme, C. , Marquet, P. , Martin, J. C. , Pickering, G. , Ardid, D. , Eschalier, A. , Dubray, C. , & Eschalier, A. (2012). Functional dysbiosis within the gut microbiota of patients with constipated‐irritable bowel syndrome. Alimentary Pharmacology & Therapeutics, 35(7), 828–838. 22315951 10.1111/j.1365-2036.2012.05007.x

[fsn34477-bib-0007] Codling, C. , O'Mahony, L. , Shanahan, F. , Quigley, E. M. , & Marchesi, J. R. (2010). A molecular analysis of fecal and mucosal bacterial communities in irritable bowel syndrome. Digestive Diseases and Sciences, 55, 392–397. 19693670 10.1007/s10620-009-0934-x

[fsn34477-bib-0008] Dallal, M. S. , Zamaniahari, S. , Davoodabadi, A. , Hosseini, M. , & Rajabi, Z. (2017). Identification and characterization of probiotic lactic acid bacteria isolated from traditional Persian pickled vegetables. GMS Hygiene and Infection Control, 12, Doc15. 10.3205/dgkh000300 PMC5627144 28989854

[fsn34477-bib-0009] El‐Salhy, M. , Gundersen, D. , Gilja, O. H. , Hatlebakk, J. G. , & Hausken, T. (2014). Is irritable bowel syndrome an organic disorder? World Journal of Gastroenterology, 20(2), 384–400. 24574708 10.3748/wjg.v20.i2.384 PMC3923014

[fsn34477-bib-0010] Emerenini, E. , Afolabi, O. , Okolie, P. , & Akintokun, A. (2013). Isolation and molecular characterization of lactic acid bacteria isolated from fresh fruits and vegetables using nested PCR analysis. British Microbiology Research Journal, 3(3), 368–377.

[fsn34477-bib-0011] Fadgyas‐Stanculete, M. , Buga, A.‐M. , Popa‐Wagner, A. , & Dumitrascu, D. L. (2014). The relationship between irritable bowel syndrome and psychiatric disorders: From molecular changes to clinical manifestations. Journal of Molecular Psychiatry, 2(1), 1–7. 25408914 10.1186/2049-9256-2-4 PMC4223878

[fsn34477-bib-0012] Ford, A. C. , Harris, L. A. , Lacy, B. E. , Quigley, E. M. , & Moayyedi, P. (2018). Systematic review with meta‐analysis: The efficacy of prebiotics, probiotics, synbiotics and antibiotics in irritable bowel syndrome. Alimentary Pharmacology & Therapeutics, 48(10), 1044–1060. 30294792 10.1111/apt.15001

[fsn34477-bib-0013] Gomathi, S. , Sasikumar, P. , Anbazhagan, K. , Sasikumar, S. , Kavitha, M. , Selvi, M. , & Selvam, G. S. (2014). Screening of indigenous oxalate degrading lactic acid bacteria from human faeces and south Indian fermented foods: Assessment of probiotic potential. The Scientific World Journal, 2014, 648059. 24723820 10.1155/2014/648059 PMC3956639

[fsn34477-bib-0014] Greco, M. , Mazzette, R. , De Santis, E. P. L. , Corona, A. , & Cosseddu, A. (2005). Evolution and identification of lactic acid bacteria isolated during the ripening of Sardinian sausages. Meat Science, 69(4), 733–739. 22063151 10.1016/j.meatsci.2004.11.004

[fsn34477-bib-0015] Harris, L. A. , & Baffy, N. (2017). Modulation of the gut microbiota: A focus on treatments for irritable bowel syndrome. Postgraduate Medicine, 129(8), 872–888. 28936910 10.1080/00325481.2017.1383819

[fsn34477-bib-0016] Herndon, C. C. , Wang, Y. P. , & Lu, C. L. (2020). Targeting the gut microbiota for the treatment of irritable bowel syndrome. The Kaohsiung Journal of Medical Sciences, 36(3), 160–170. 31782606 10.1002/kjm2.12154 PMC11896346

[fsn34477-bib-0017] Kassinen, A. , Krogius‐Kurikka, L. , Mäkivuokko, H. , Rinttilä, T. , Paulin, L. , Corander, J. , Malinen, E. , Apajalahti, J. , & Palva, A. (2007). The fecal microbiota of irritable bowel syndrome patients differs significantly from that of healthy subjects. Gastroenterology, 133(1), 24–33. 17631127 10.1053/j.gastro.2007.04.005

[fsn34477-bib-0018] Khalil, R. , El‐Halafawy, K. , Mahrous, H. , Kamaly, K. , Frank, J. , & El Soda, M. (2007). Evaluation of the probiotic potential of lactic acid bacteria isolated from faeces of breast‐fed infants in Egypt. African Journal of Biotechnology, 6(7), 939–949.

[fsn34477-bib-0019] Kim, Y. S. , & Kim, N. (2018). Sex‐gender differences in irritable bowel syndrome. Journal of Neurogastroenterology and Motility, 24(4), 544–558. 30347934 10.5056/jnm18082 PMC6175559

[fsn34477-bib-0020] Kosako, M. , Akiho, H. , Miwa, H. , Kanazawa, M. , & Fukudo, S. (2018). Impact of symptoms by gender and age in Japanese subjects with irritable bowel syndrome with constipation (IBS‐C): A large population‐based internet survey. BioPsychoSocial Medicine, 12(1), 1–11. 30186363 10.1186/s13030-018-0131-2 PMC6122187

[fsn34477-bib-0021] Kumar, S. , Pattanaik, A. K. , Sharma, S. , Jadhav, S. E. , Dutta, N. , & Kumar, A. (2017). Probiotic potential of a *Lactobacillus* bacterium of canine faecal‐origin and its impact on select gut health indices and immune response of dogs. Probiotics and Antimicrobial Proteins, 9, 262–277. 28188477 10.1007/s12602-017-9256-z

[fsn34477-bib-0022] Lacy, B. E. , & Patel, N. K. (2017). Rome criteria and a diagnostic approach to irritable bowel syndrome. Journal of Clinical Medicine, 6(11), 99. 29072609 10.3390/jcm6110099 PMC5704116

[fsn34477-bib-0023] Liu, H.‐N. , Wu, H. , Chen, Y.‐Z. , Chen, Y.‐J. , Shen, X.‐Z. , & Liu, T.‐T. (2017). Altered molecular signature of intestinal microbiota in irritable bowel syndrome patients compared with healthy controls: A systematic review and meta‐analysis. Digestive and Liver Disease, 49(4), 331–337. 28179092 10.1016/j.dld.2017.01.142

[fsn34477-bib-0024] Ljungh, A. , & Wadstrom, T. (2006). Lactic acid bacteria as probiotics. Current Issues in Intestinal Microbiology, 7(2), 73–90. 16875422

[fsn34477-bib-0025] Mättö, J. , Maunuksela, L. , Kajander, K. , Palva, A. , Korpela, R. , Kassinen, A. , & Saarela, M. (2005). Composition and temporal stability of gastrointestinal microbiota in irritable bowel syndrome – A longitudinal study in IBS and control subjects. FEMS Immunology and Medical Microbiology, 43(2), 213–222. 15747442 10.1016/j.femsim.2004.08.009

[fsn34477-bib-0026] Menconi, A. , Kallapura, G. , Latorre, J. D. , Morgan, M. J. , Pumford, N. R. , Hargis, B. M. , & Tellez, G. (2014). Identification and characterization of lactic acid bacteria in a commercial probiotic culture. Bioscience of Microbiota, Food and Health, 33(1), 25–30. 24936379 10.12938/bmfh.33.25 PMC4034328

[fsn34477-bib-0027] Mikelsaar, M. , Sepp, E. , Štšepetova, J. , Songisepp, E. , & Mändar, R. (2016). Biodiversity of intestinal lactic acid bacteria in the healthy population. Advances in Microbiology, Infectious Diseases and Public Health, 4, 1–64. 10.1007/5584_2016_3 27167411

[fsn34477-bib-0028] Moraes‐Filho, J. P. , & Quigley, E. M. (2015). The intestinal microbiota and the role of probiotics in irritable bowel syndrome: A review. Arquivos de Gastroenterologia, 52, 331–338. 26840477 10.1590/S0004-28032015000400015

[fsn34477-bib-0029] Mulaw, G. , Sisay Tessema, T. , Muleta, D. , & Tesfaye, A. (2019). In vitro evaluation of probiotic properties of lactic acid bacteria isolated from some traditionally fermented Ethiopian food products. International Journal of Microbiology, 2019, 7179514. 31534458 10.1155/2019/7179514 PMC6732631

[fsn34477-bib-0030] Ni, K. , Wang, Y. , Li, D. , Cai, Y. , & Pang, H. (2015). Characterization, identification and application of lactic acid bacteria isolated from forage paddy rice silage. PLoS One, 10(3), e0121967. 25803578 10.1371/journal.pone.0121967 PMC4372580

[fsn34477-bib-0031] Noor, S. O. , Ridgway, K. , Scovell, L. , Kemsley, E. K. , Lund, E. K. , Jamieson, C. , Johnson, I. T. , & Narbad, A. (2010). Ulcerative colitis and irritable bowel patients exhibit distinct abnormalities of the gut microbiota. BMC Gastroenterology, 10(1), 1–9. 21073731 10.1186/1471-230X-10-134 PMC3002299

[fsn34477-bib-0032] Pelinescu, D. , Chifiriuc, M. , Ditu, L. , Sarbu, I. , Bleotu, C. , Vassu, T. , Stoica, I. , Lazar, V. , Corcionivoschi, N. , Sasarman, E. , & Sasarman, E. (2011). Selection and characterization of the probiotic potential of some lactic acid bacteria isolated from infant feces. Romanian Biotechnological Letters, 16(3), 6178–6189.

[fsn34477-bib-0033] Rodiño‐Janeiro, B. K. , Vicario, M. , Alonso‐Cotoner, C. , Pascua‐García, R. , & Santos, J. (2018). A review of microbiota and irritable bowel syndrome: Future in therapies. Advances in Therapy, 35, 289–310. 29498019 10.1007/s12325-018-0673-5 PMC5859043

[fsn34477-bib-0034] Růžičková, M. , Vítězová, M. , & Kushkevych, I. (2020). The characterization of *Enterococcus* genus: Resistance mechanisms and inflammatory bowel disease. Open Medicine, 15(1), 211–224. 32292819 10.1515/med-2020-0032 PMC7147287

[fsn34477-bib-0035] Sulistiani, S. , Abinawanto, A. , Sukara, E. , Salamah, A. , Dinoto, A. , & Mangunwardoyo, W. (2014). Identification of lactic acid bacteria in sayur asin from Central Java (Indonesia) based on 16S rDNA sequence. International Food Research Journal, 21(2), 527–532.

[fsn34477-bib-0036] Tamura, K. , Stecher, G. , & Kumar, S. (2021). MEGA11: Molecular evolutionary genetics analysis version 11. Molecular Biology and Evolution, 38(7), 3022–3027. 33892491 10.1093/molbev/msab120 PMC8233496

[fsn34477-bib-0037] Tap, J. , Derrien, M. , Törnblom, H. , Brazeilles, R. , Cools‐Portier, S. , Doré, J. , Störsrud, S. , Le Nevé, B. , Öhman, L. , & Simrén, M. (2017). Identification of an intestinal microbiota signature associated with severity of irritable bowel syndrome. Gastroenterology, 152(1), 111–123. 27725146 10.1053/j.gastro.2016.09.049

[fsn34477-bib-0038] Tinrat, S. , Saraya, S. , & Chomnawang, M. T. (2011). Isolation and characterization of *Lactobacillus salivarius* MTC 1026 as a potential probiotic. The Journal of General and Applied Microbiology, 57(6), 365–378. 22353742 10.2323/jgam.57.365

[fsn34477-bib-0039] Wang, X. , Wang, W. , Lv, H. , Zhang, H. , Liu, Y. , Zhang, M. , Wang, Y. , & Tan, Z. (2021). Probiotic potential and wide‐spectrum antimicrobial activity of lactic acid bacteria isolated from infant feces. Probiotics and Antimicrobial Proteins, 13, 90–101. 32405962 10.1007/s12602-020-09658-3

[fsn34477-bib-0040] Wood, B. (1992). The Lactic Acid Bacteria. volume 1.

[fsn34477-bib-0041] Zhu, Y. , Luo, T. M. , Jobin, C. , & Young, H. A. (2011). Gut microbiota and probiotics in colon tumorigenesis. Cancer Letters, 309(2), 119–127. 21741763 10.1016/j.canlet.2011.06.004 PMC3148272

[fsn34477-bib-0042] Zhuang, X. , Tian, Z. , Li, L. , Zeng, Z. , Chen, M. , & Xiong, L. (2018). Fecal microbiota alterations associated with diarrhea‐predominant irritable bowel syndrome. Frontiers in Microbiology, 9, 1600. 30090090 10.3389/fmicb.2018.01600 PMC6068233

